# Exposure to secondary traumatic stress and its related factors among emergency nurses in Saudi Arabia: a mixed method study

**DOI:** 10.1186/s12912-024-02018-4

**Published:** 2024-05-18

**Authors:** Bushra Alshammari, Nada F Alanazi, Fatmah Kreedi, Farhan Alshammari, Sameer A. Alkubati, Awatif Alrasheeday, Norah Madkhali, Ammar Alshara, Venkat Bakthavatchaalam, Mahmoud Al-Masaeed, Sabah Kaied Alshammari, Nwair Kaied Alshammari, Mukhtar Ansari, Arshad Hussain, Ahmed K. Al-Sadi

**Affiliations:** 1 Medical Surgical Nursing Department, College of Nursing, University of Hail, Hail, 2440 Saudi Arabia; 2 Hail General Hospital, Hail Health Cluster, Hail, Saudi Arabia; 3 Public Authority of Disabled, Kuwait City, 34R5+25Q 212 Kuwait; 4 Department of Pharmaceutics, College of Pharmacy, University of Hail, Hail, 2440 Saudi Arabia; 5 Department of Nursing, Faculty of Medicine and Health Sciences, Hodeida University, Hodeida, Yemen; 6 Nursing Administration Department, College of Nursing, University of Hail, Hail, Saudi Arabia; 7 Department of Nursing, College of Nursing, Jazan University, Jazan, 45142 Saudi Arabia; 8 School of Engineering, Cardiff University, Cardiff Wales, UK; 9 Faculty of Medicine and Health Sciences, Faculty of Health and Medicine, University Putra Malaysia, University of Newcastle, Serdang, Selangor Malaysia; 10 Faculty of Health and Medicine, University of Newcastle, Callaghan, 2308 Australia; 11 Total quality and patient safety department, King Salman Specialist Hospital- Cardiac Center, Hail Health Cluster, Hail, Saudi Arabia; 12 Outpatient department, King Salman Specialist Hospital- Cardiac Center, Hail Health Cluster, Hail, Saudi Arabia; 13 Department of Clinical Pharmacy, College of Pharmacy, University of Hail, Hail, Saudi Arabia

**Keywords:** ED nurses, Stress, STS, STSS, Secondary traumatic stress, Socio-demographic

## Abstract

**Background:**

Emergency department (ED) nurses are exposed to the risk of secondary traumatic stress (STS), which poses a threat not only to nurses’ health and psychological well-being but also adversely affects the execution of their professional duties. The quality and outcome of their nursing services are negatively affected by STS.

**Purpose:**

The purpose of this study is to comprehensively investigate the prevalence and intensity of Secondary Traumatic Stress (STS) among Emergency Department (ED) nurses. It aims to identify and analyze the socio-demographic, occupational, and psychological factors that influence the severity and variation of STS experienced by these nurses.

**Methods:**

The study utilized a sequential explanatory mixed methods approach, including two phases. Phase 1 employed a cross-sectional study design, utilizing a convenience sample of 181 nurses to explore the levels of STS and the factors associated with it. Following this, Phase 2 was structured as a qualitative descriptive study, which involved conducting semi-structured interviews with a purposefully selected group of ten ED nurses. Data collection took place at three major hospitals in Saudi Arabia during the period from January to June 2022.

**Results:**

A total of 181 participants were included in the study. The mean STSS score reported by the nurses was 51 (SD = 13.23) out of the maximum possible score of 85, indicating severe STS among ED nurses. Factors associated with an increase in the levels of STS among ED nurses included being female, older in age, married, possessing higher education and experience, having a positive relationship with colleagues, receiving organisational support, and dealing with a higher number of trauma cases. Several themes emerged from the qualitative interviews including: ED Characteristics: Dual Impact on STS, Emotional Resonance and Vulnerability, Personal Life Stressors, The Ability to Cope, and Social Support.

**Conclusion and implications for practice:**

Future strategies and interventions targeting STS should be prioritized to effectively manage its impact on ED nurses. It is crucial to develop targeted interventions that address the specific factors contributing to STS, as identified in this study. Additionally, these findings aim to enhance awareness among nursing administrators, managers, and supervisors about the critical factors associated with STS. This awareness is essential for accurately assessing and developing interventions that mitigate STS among nursing staff.

**Supplementary Information:**

The online version contains supplementary material available at 10.1186/s12912-024-02018-4.

## Background

Nurses play a pivotal role in delivering healthcare services, often serving as the primary the primary and main point of contact between patients and healthcare providers [1, 2]. They spend most of their working time directly relating to and interacting with patients. It is crucial to ensure that the nurses’ welfare is supported to enhance their professional development, quality of work, and output levels. Emergency Department (ED) nurses, in particular, work in highly demanding environments [3], where the intensity of work and the level of effort and empathy required are significantly higher. In this unit, the nurses work with patients who are, in most instances, unable to execute their basic hygienic needs and duties [4]. Frequently, ED nurses provide care for patients who have experienced traumatic events, such as accidents and injuries, and wounded and haemorrhaging victims. A majority of ED patients are traumatised by their experiences and often share this with ED nurses. As such, on a regular basis, ED nurses work and serve patients with trauma, which exposes them to the risk of trauma [5, 6]. The Diagnostic and Statistical Manual of Mental Disorders, 5th Edition (DSM-5) has broadened the definition of trauma to include indirect exposure to trauma—hearing about, witnessing, and learning about trauma—through indirect means [7, 8]. Thus, trauma refers not only to direct trauma from an assault but also to secondary exposure to trauma. The re-conceptualisation of trauma leads to the recognition of secondary traumatic stress (STS) as a form of traumatic stress in addition to post-traumatic stress disorder (PTSD) diagnosis. PTSD is a mental health problem that occurs in people after they encounter a life-threatening experience [8, 9]. With both PTSD and STS characterized by symptoms of intrusion, avoidance, and arousal [7]. In the period preceding DSM-5, individuals were only diagnosed with PTSD after prolonged exposure to trauma, which limited the number of people diagnosed. Many were incorrectly diagnosed as not having PTSD. The most prevalent risk of trauma exposure for ED nurses is STS [7, 8]. STS is the result of stress caused by indirect trauma exposure. This stress dimension is acquired secondarily. The primary stress is experienced by patients who have been exposed to a traumatic event. In turn, the secondary level of stress affects the nurses who care for these patients. Nurses have a responsibility to engage patients while offering care. This includes taking their medical histories and understanding the context and nature of their injuries and accidents. Consequently, they often gather information on patients’ traumatic experiences. This exposure to patients’ traumatic stories and histories can lead to nurses experiencing STS. Understanding the level and exposure of nurses to STS is critical as its prevalence affects their psychosocial wellness and quality performance [10, 11].

In understanding the prevalence of STS among nurses, studies have demonstrated a correlation between the nurses’ socio-demographic factors and their STS levels. However, these factors are inherently contextual. ED nurses encounter a variety of socio-demographic factors across different regions and countries [12, 13]. Therefore, findings the relationship between socio-demographic factors and STS levels depend on variables such as health policies, cultural influences, and professional expectations within each country and region [12, 13]. Thus, findings are formulated by analyzing dataspecific to each region and country. Unfortunately, preliminary literature analysis in the Saudi context demonstrated limited data on social factors and job satisfaction among Saudi Arabian nurses [6]. This gap in the literature guided the study’s focus on primary data collection within the Saudi Arabian context. The study was developed based on the KSA public sector healthcare industry context. Thus, the focus was on nurses working in the public healthcare industry. This focus was chosen because the public healthcare sector constitutes over two-thirds of the KSA healthcare industry. An evaluation of the KSA context indicates high exposure to STS among its nurses.

The level of STS in the Middle East is higher than in the global average. For example, studies by Kinker, Arfken and Morreale [14] and Shalabi et al. [15] which used the STSS tool, have shown that nurses in the Middle East experience greater exposure to STS compared to their counterparts in Western Europe and globally. In the Middle East, cultural perceptions often view stress, depression, and all forms of mental illness as a curse and socially unacceptable. As a result, individuals facing such challenges are often ostracized, viewed as insane, and considered unfit for society. This stigma significantly increases the likelihood of individuals not seeking help, treatment, and care when they are exposed to STS. Furthermore, seeking psychiatric assistance or counselling for traumatic experiences is frequently seen as an admission of mental instability, thus discouraging many from seeking such help [16]. This distinct cultural context makes the Middle East an especially relevant location for a study aimed at examining the impact of these perceptions on STS levels and exposure factors.

The strategic aim and contribution of the study is to help evaluate the cause of the relatively high STS among nurses in KSA. Specifically, the study aims to determine if the contributing factors and the extent of exposure to STS in KSA are consistent with those identified in the global literature. This provides a foundational basis for developing effective strategies to overcome and mitigate STS among nurses in KSA. By understanding these factors, employees and organizations can devise strategic and practical solutions to alleviate STS and reduce exposure among public sector nurses in KSA. Organizations will benefit from having more positive, committed, and productive employees, while also reducing costs associated with stress-related issues [17].

### Justification for conducting a mixed methods approach

The Mixed Methods Approach allows for a more comprehensive understanding of the complex relationship between socio-demographic and work-related factors and STS. Quantitative methods can identify and measure the extent of these relationships through statistical analysis, while qualitative methods can provide deeper insights into the experiences and perceptions of ED nurses regarding STS. Qualitative findings can also validate the results obtained from quantitative methods. Given the cultural context of Saudi Arabia, qualitative methods can explore cultural factors that might influence STS. These insights are crucial for tailoring interventions and policies effectively.

### Study aim

Based on the identified literature gap, this study aimed to comprehensively assess the prevalence and intensity of STS among nurses working in the ED. Additionally, the study aims to identify and analyze the specific socio-demographic, occupational, and psychological factors that contribute to the variation in STS levels among these nurses.

## Materials and design

### Research design

The research utilized a mixed methods sequential explanatory approach, commencing with a quantitative phase followed by a qualitative phase [18]. Phase 1: used a cross-sectional design to measure the prevalence of STS among ED nurses and the nature and extent of the relationship between ED nurses’ STS levels and their socio-demographic and work-related variables [19]. Phase 2 involved a qualitative descriptive approach, which included conducting several semi-structured interviews. These interviews were designed to enhance the understanding of the Phase 1 findings by providing a context in which the quantitative data can be better interpreted [18]. Qualitative interviews helped in gaining deeper insights into the lived experiences of individuals dealing with traumatic stress and in exploring the various factors that impact the levels of stress among nurses. Both qualitative and quantitative data were gathered and subsequently integrated to offer a comprehensive understanding of the experience of STS and how various predictors contributed to an increase in its levels.

### Setting and participants

This study was conducted from January to June 2022. Phase 1 of the study used a convenience sample of ED nurses recruited from three selected governmental hospitals in Saudi Arabia: Hail General Hospital, King Khalid Hospital, and King Salman Specialist Hospital. A sample size of 181 ED nurses was determined using OpenEpi web-based calculator, Version 3.01 (www.openepi.com) based on the following criteria: 95% confidence level, 5% absolute precision and a population size of 340. The inclusion criterion was that the participant must be currently a registered nurse who provides direct patient care in an ED in the targeted hospitals and agreed to participate in the study. Moreover, nurses who had more than one year of experience in the ED were included. Trainees were excluded from the study.

In Phase 2 of the study, we interviewed a purposeful sample of 10 nurses who had both higher and lower scores on the STSS. Choosing nurses with varying stress scores helped understand factors contributing to higher or lower STS levels, leading to more precise research outcomes relevant to the context. Interviews were carried out until data saturation was reached, where no additional themes or subthemes were found by the participants [20]. When the terms and processes started to repeat, it indicates that a sufficient amount of data has been collected [21]. Each interview lasted for approximately 30 to 60 min.

### Data collection

#### Questionnaires

The questionnaire has two sections which include collecting participants’ socio-demographic characteristics, such as age, gender, ethnicity, marital status, education, experience, dependents, and income. It also gathered information on factors like career rank, shift work, weekly hours, spirituality, personal trauma history, trauma caseload, organizational support, and colleague relationships.

The second section of the questionnaire assessed STS using the English validated version of the Secondary Traumatic Stress Scale (STSS) developed by Bride et al. [22]. Since English is the official language among nurses in the intended settings, this tool was chosen. The STSS is a well-established tool with proven reliability, characterized by the Cronbach’s alpha value of 0.89 [23]. The tool includes a total of 17 different questions that measure stress using five-point, self-rating scales with responses ranging from 1 to 5, with 1 = never and 5 = very often. The questions are clustered into the three elements of STS: (i) intrusion (questions 2, 3, 6, 10, and 13), (ii) arousal (questions 4, 8, 11, 15, and 16), and (iii) avoidance (questions 1, 5, 7, 9, 12, 14, and 17) [24, 25]. In its assessment of stress levels, the questionnaire focuses on the respondents’ experiences in the last seven days. The scores range between 17 and 85, with the higher scores indicating higher levels of STS. The STSS scores have the following interpretation: <28 indicating little or no STS, 28–37 indicating mild STS, 38–43 indicating moderate STS, 44–48 indicating high STS, and 49 and above indicating severe STS [24, 26].

The responses were collected online via Google forms, as the study questionnaire was published online, and the respondents accessed it through a URL link that was shared with them. The questionnaire was distributed by ED directors to the nurses who met the inclusion criteria. Moreover, the questionnaire had an attached consent with a brief clarification of the study purposes and a number to contact in case of any questions. The questionnaire included an empty field where participants could indicate wish to be contacted and their preferred method of communication if they wanted to participate in the second phase of the study. The data were collected in the period between January and June 2022, thereby providing the respondents with sufficient enough time to respond to the questionnaire in the midst of their busy and tight working schedules. A reminder to complete the questionnaire was sent three weeks after the first attempt to increase response rates.

#### Interviews

In-depth semi-structured qualitative interviews were conducted with a purposeful sample of nurses who had participated in Phase 1. The researcher used SPSS to identify and recruit nurses with the highest and lowest scores on the STSS. If these nurses did not provide their contact information or express interest in participating in the first phase, the researcher would then proceed to recruit participants with the next highest and lowest scores on the STSS.

Participants were contacted and given an information sheet that detailed the purpose and nature of the interviews, along with the consent process. Subsequently, the researcher and study participants convened at a mutually agreed-upon private venue for the interview sessions, involving only the researcher and the participant, while some interviews were conducted over the telephone as per the participants’ preferences.

The interview guide (Supplementary 1) was created and developed by the researchers following the initial analysis of Phase 1 and a review of relevant literature. The guide was used to capture the experiences of nurses in relation to STS while they cared for patients admitted to the ED. The following questions were asked: Can you describe a specific incident or situation in your nursing practice that you found particularly stressful or emotionally challenging? What factors or things could exacerbate or alleviate the traumatic stress that you experience? Are there any specific factors or aspects of your work environment that you believe contribute to higher or lower levels of STS (explain)? What do you think could be done to improve the well-being and mental health of nurses who frequently encounter STS? Can you recall a moment when you felt overwhelmed by STS? How did you handle it, and what support did you seek or receive? How do you manage or deal with STS in your professional capacity? The interviewer proceeded to ask further open-ended questions that were customized based on each participant’s specific responses and experiences.

The interview notes incorporated observations of participants’ body language and emotions which were also used during subsequent data analysis. Interviews were recorded using audio in a quiet and comfortable room that allowed individuals to freely express themselves without disturbances.

### Ethical considerations

#### Ethical approval

for the study was obtained from the Institutional Review Board (IRB), represented by the Health Cluster in Hail city (registered with the King Abdullah City for Science and Technology (KACST) in the KSA, under the registration number H-08-L-074, with approval reference H-2022-20. All the participants in this study were informed about the purpose of the study and its advantages before being asked to fill out the questionnaire. In addition, autonomy to participate in the study was guaranteed, and all information was kept confidential and used only for the purpose of scientific research. Anonymity was assured by using anonymous surveys that cannot be traced back to the respondent. The survey contained no personally identifiable information such as name or contact information. All responses were gathered and combined together and summarized in the report to further protect participants anonymity.

### Data analysis

#### Phase 1: a cross sectional study

The analysis approach included the use of a statistical analysis process. The study’s analysis process relied on the use of SPSS (version 26) software. In the analysis process, the findings were categorised into two main levels: the descriptive and the inferential statistics analysis. First, the descriptive analysis process enabled the analysis of the study sample–based demographics. The socio-demographic variables of the ED nurses were analyzed descriptively with the use of frequency and percentages to indicate the representation of the different population segments. Furthermore, the prevalence of the STSS variables and the presence of PTSD among the ED nurses were both descriptively analysed through the use of mean and standard deviation variables. Additionally, the study checked for the normality of the distributions using the Kolmogorov–Smirnov test and illustrated that p value greater than 0.05 indicates normal distribution of the data. Therefore, parametric statistics tests were used in this study.

Then, an independent-samples Student’s t-test was utilized to test the relationship between the STSS scores and the two categorical variables while one-way analysis of variance (ANOVA) was used to test the relationship between the STSS scores and three or more categorical variables. The obtained findings were presented in tables to ease the understanding and interpretation for readers. Factors that appear to have a statistically significant association with STSS scores were then analysed to identify the independent factors of ED nurses’ STSS using multiple linear regression. A p-value of ˂0.05 was considered statistically significant.

#### Phase 2: qualitative descriptive design

Thematic analysis was employed to analyze the interviews [27]. Coding was managed using NVivo qualitative data analysis software Version 12 [28]. In our qualitative analysis, we employed a structured three-phase approach: data reduction, data display, and conclusion drawing/verification [29]. . Initially, the research team conducted a detailed review of all interview transcripts, applying line-by-line coding to highlight significant phrases and identify emerging patterns. This process was enhanced by independent coding by two team members, ensuring data reliability through consensus on code assignment. During the data display phase, we organized the coded data using matrices and diagrams, which facilitated the examination of relationships and the comparison of themes across the dataset. This visual organization helped refine codes into more focused categories. In the final phase, we synthesized the data to draw meaningful conclusions, ensuring our interpretations were grounded in the participants’ experiences. Member checking was employed to validate our findings, further bolstering the credibility of our analysis. To ensure interpretative accuracy, maintain reliability, and bolster rigor, the findings were methodically discussed and validated with colleagues at every stage of the research process [30].

### Rigor

The Good Reporting of a Mixed Methods Study (GRAMMS) guidelines were utilized to improve the quality and transparency of the study [31]. Interviews were transcribed and independently coded by three team members (BA, FK, and FA) for dependability and confirmability. Emerging codes and themes were collectively discussed and agreed upon [32]. Member verification was carried out throughout the interview process.

## Results

### Quantitative results

#### Demographic findings and sample validity

A total of 181 nurses completed the questionnaire. The first findings analyzed in the study focused on the sample demographic variables as illustrated in Table 1. Overall, 50.8% (*n* = 92) of the total participants identified themselves as being female, with 49.2% (*n* = 89) as being male. The average age of these participants was 29.9 years, ranging between 20 and 46. The majority, at 80.1% (*n* = 145), were identified as Arabs. In terms ofmarital status, 56.4% (*n* = 102) were unmarried, while 43.6% (*n* = 79) were married. Professionally, 26% had a diploma education level, while 58% and 16% Held at least a bachelor and master’s degree qualification, respectively. On earnings, the majority earn between 5000 and 10,000 Saudi Riyal (SAR) at 37%, with only 16.6% reporting to earn more than 15,000 SAR monthly income salary.

**Table 1:** Demographic characteristics of participants (*n* = 181)

Variable	Frequency	Percent
**Age** Mean (SD)	29.9 (5.5)	
**Gender**		
Male	89	49.2
Female	92	50.8
**Ethnicity**		
Arabs	145	80.1
Filipino	20	11.0
Indian	16	8.8
**Years of experience** Mean (SD)	7.6 (5.3)	
**Marital Status**		
Unmarried	102	56.4
Married	79	43.6
**Dependents**		
No dependent	10	5.5
1–2	84	46.4
3–4	58	32.0
more than 4	29	16.0
**Education**		
Diploma	47	26.0
Bachelor’s Degree	105	58.0
Master’s Degree	29	16.0
**Monthly Income (in SAR)**		
Less than 5000	36	19.9
5000-10,000	67	37.0
10,000–15,000	48	26.5
More than 15,000	30	16.6

SD; Standard deviation, SAR; Saudi Riyal

#### STSS scoring among participants

**Fig. 1:**
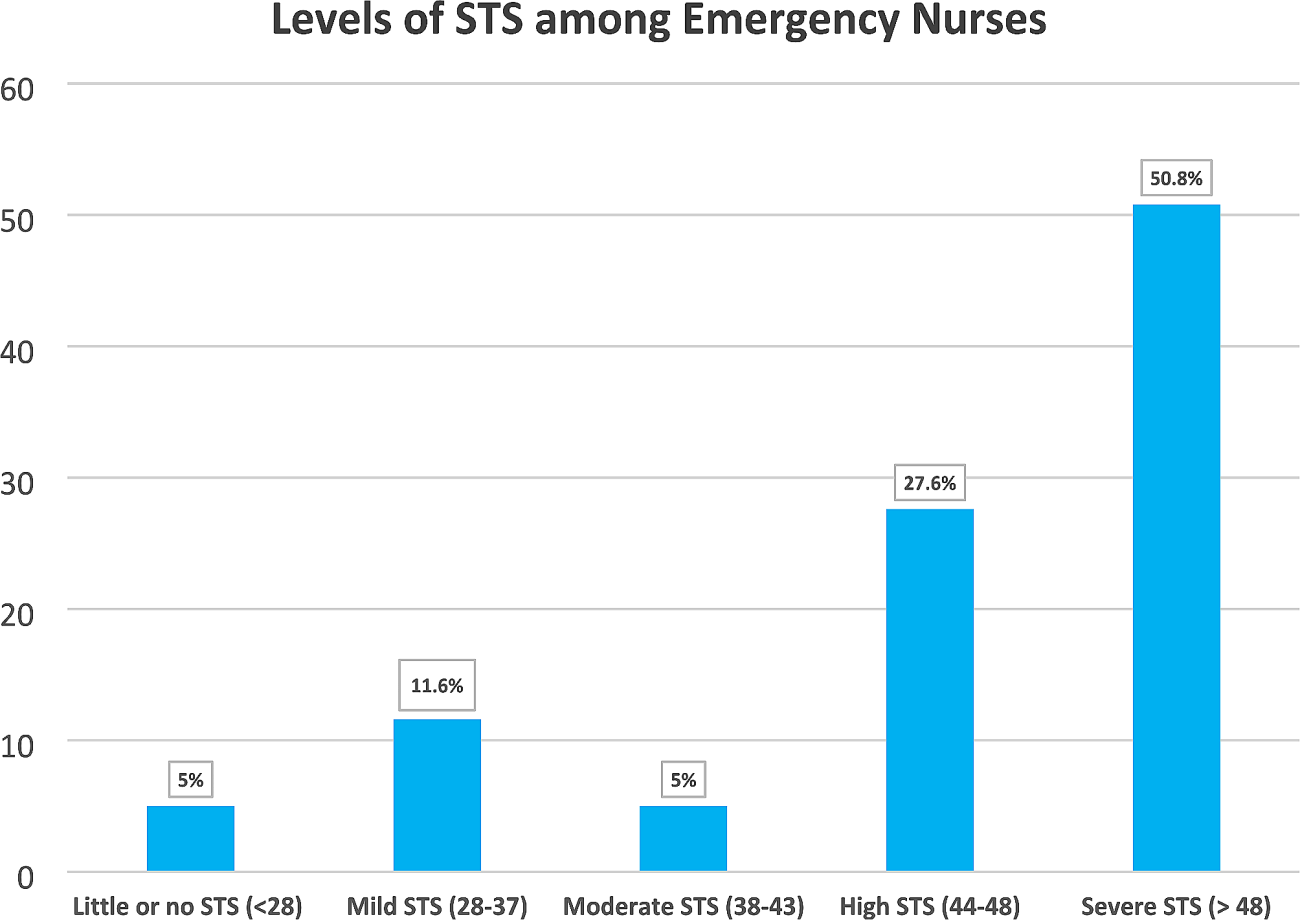
Levels of STS reported by ED nurses

This study analysed the level of STS among ED nurses. The analysis relied on the scores derived from participants’ responses to 17 questions. According to STSS, the mean STS score reported by the nurses was 51.0 (SD = 13.2) out of a possible score of 85, thereby indicating severe STS among the ED nurses. A small proportion of participants (5%) reported experiencing Little to no, or moderate STS, whereas 11.6% indicated mild STS. The majority of participants disclosed experiencing high and severe levels of STS, with 27.6% reporting high levels and 50.8% reporting severe levels. Figure 1 displays the distribution of STS levels among ED nurses.

#### Scoring of STSS subscales: intrusion, arousal, and avoidance variables

The STSS scoring examined the respective scores of the three elements of STS, namely intrusion, arousal, and avoidance. Table 2 outlines the average mean for the three elements of STSS and for the total score of STSS of the respondents in the study. For the three different STSS subscales, the analysis established that out of the highest possible score of 35, avoidance symptoms had the highest score of 20.62 (SD = 5.87), followed by the intrusion with a mean score of15.57 (SD = 3.97), and arousal with a mean score of 14.80 (SD = 4.38).

**Table 2:** Mean scores for the three subscales of STSS

Descriptive Statistics			
	N	Minimum- Maximum	M ± SD
Intrusion	181	5.00–25.00	15.57 ± 3.97
Avoidance	181	7.00–35.00	20.62 ± 5.87
Arousal	181	5.00–25.00	14.80 ± 4.38
Total STSS	181	17.00–85.00	51.00 ± 13.23

M: mean; SD: standard deviation; STSS: secondary traumatic stress scale

#### STS symptoms as reported by ED nurses

The most frequently reported avoidance symptoms included a perceived foreshortened future (76%), followed by diminished activity level (75%), avoidance of clients (68%), and inability to recall client information (62%) respectively. The remaining avoidance symptoms were reported less frequently, including emotional numbing (51%); avoidance of people, places, and things (53%), and detachment from others (56%). Among intrusion symptoms, the most commonly reported symptoms were cued psychological distress (79%), disturbing dreams about clients (69%), and sense of reliving clients’ trauma (63%) while the remaining intrusion symptoms were reported less frequently. Regarding arousal symptoms, the majority of ED nurses indicated experiencing difficulty sleeping (76%), hypervigilance (71%) and irritability (70%). Table 3 illustrates the prevalence of STS symptoms among ED nurses.

**Table 3:** Frequency and percentages of STS symptoms reported by ED nurses

	Never	Rarely	Occasionally	Often	Very often
1. Emotionally numb.	47 (26.0%)	43 (23.8%)	44 (24.3%)	34 (18.8%)	13 (7.2%)
2. Cued physiological reaction	10 (5.5%)	69 (38.1%)	53 (29.3%)	32 (17.7%)	17 (9.4%)
3. Sense of reliving clients’ trauma	16 (8.8%)	51 (28.2%)	50 (27.6%)	33 (18.2%)	31 (17.1%)
4. Difficulty sleeping	25 (13.8%)	19 (10.5%)	50 (27.6%)	65 (35.9%)	22 (12.2%)
5. Foreshortened future	22 (12.2%)	21 (11.6%)	84 (46.4)	31 (17.1%)	23 (12.7%)
6. Cued psychological distress	10 (5.5%)	29 (16.0%)	32 (17.7%)	55 (30.4%)	55 (30.4%)
7. Detachment from others	14 (7.7%)	65 (35.9%)	51 (28.2%)	34 (18.8%)	17 (9.4%)
8. Easily startled	21 (11.6%)	72 (39.8%)	36 (19.9%)	34 (18.8%)	18 (9.9%)
9. Diminished activity level	19 (10.5%)	27 (14.9%)	53 (29.3%)	68 (37.6%)	14 (7.7%)
10. Intrusive thoughts about clients	22 (12.2%)	69 (38.1%)	48 (26.5%)	28 (15.5%)	14 (7.7%)
11. Difficulty concentrating	30 (16.6%)	70 (38.7%)	35 (19.3%)	33 (18.2%)	13 (7.2%)
12. Avoidance of people, places, and things	26 (14.4%)	59 (32.6%)	39 (21.5%)	40 (22.1%)	17 (9.4%)
13. Disturbing dreams about clients	29 (16.0%)	28 (15.5%)	33 (18.2%)	40 (22.1%)	51 (28.2%)
14. Avoidance of clients	20 (11.0%)	38 (21.0%)	60 (33.1%)	38 (21.0%)	25 (13.8%)
15. Irritability	19 (10.5%)	36 (19.9%)	76 (42.0%)	30 (16.6%)	20 (11.0%)
16. Hypervigilance	33 (18.2%)	20 (11.0%)	23 (12.7%)	80 (44.2%)	25 (13.8%)
17. Inability to recall client information	23 (12.7%)	46 (25.4%)	35 (19.3%)	44 (24.3%)	33 (18.2%)

#### Relationship between emergency nurses’ demographics and STSS scores

Table 4 illustrates the relationship between the ED nurses’ sociodemographic characteristics and their overall STSS scores. Significant relationship were observed between the STSS scores and the variables of age, gender, years of experience, marital status, and educational level, with p-values of 0.010, 0.001, 0.001, 0.008, and 0.003, respectively. Conversely, no significant association was found between the STSS scores and the variables of ethnicity, number of dependents, and monthly income.

**Table 4:** ED nurses’ socio-demographic factors and their exposure to secondary traumatic stress

Factor	Group	*N*	Mean ± SD	test value	*p*-value
Age	20–28	91	50.47 ± 13.72	F (4.678)	0.010*
	29–33	49	55.3061 ± 10.23		
	34–46	41	47.0488 ± 14.11		
Gender	Male	92	45.76 ± 12.01	t (5.678)	0.001*
	Female	89	56.07 ± 12.40		
Ethnicity	Arabs	145	50.56 ± 13.45	F (0.763)	0.468
	Filipino	20	51.10 ± 11.90		
	Indian	16	54.87 ± 12.92		
Years of experience	≤ 5	74	52.43 ± 13.53	F (7.575)	0.001*
	6–9	46	55.26 ± 11.18		
	˃10	61	46.06 ± 12.93		
Marital Status	Unmarried	102	48.70 ± 14.91	t (-2.703)	0.008*
	Married	79	53.97 ± 10.00		
Number of dependents	No dependent	10	41.50 ± 13.99	F (1.906)	0.130
	1–2	84	51.34 ± 14.27		
	3–4	58	51.41 ± 12.66		
	˃4	29	52.48 ± 9.84		
Educational level	Diploma	47	47.72 ± 10.54	F (6.110)	0.003*
	Bachelor’s Degree	105	53.81 ± 14.48		
	Master’s Degree	29	46.13 ± 9.55		
Monthly income (in SAR)	Less than 5000	36	48.36 ± 15.96	F (1.205)	0.310
	5000-10,000	67	52.61 ± 11.17		
	10,000–15,000	48	52.12 ± 13.51		
	More than 15,000	30	48.80 ± 13.31		

*Significant; t: Independent t test; F: ANOVA test

#### Relationship between emergency nurses’ work-related items and STSS scores

Table 5 shows that there were a significant relationships between the STSS scores and the variables of Trauma Case Load, ED nurses’ organisational Support, and their relationship with colleagues with p-value of 0.001, 0.027 and 0.026, respectively. However, no significant relationship was found between the STSS scores and other item.

**Table 5:** Emergency nurses’ work-related factors and their exposure to STS

Factor	Group	*N*	Mean ± SD	test value	*p*-value
Career Rank	Staff Nurse	107	52.03 ± 12.81	F (1.830)	0.143
	Head Nurse	26	53.57 ± 18.65		
	Assistant Nurse	21	46.85 ± 4.70		
	Supervisor	27	47.66 ± 12.41		
Shift Worked	Morning shift	61	51.04 ± 15.10	F (0.365)	0.778
	Afternoon shift	22	48.77 ± 9.81		
	Night shift	3	47.00 ± 10.53		
	All shift	95	51.62 ± 12.79		
Weekly Working Hours	Less than 40 h	44	51.81 ± 11.41	F (0.343)	0.710
	40 h	91	51.29 ± 12.39		
	More than 40 h	46	49.65 ± 16.31		
Spirituality level	Strength in faith	102	51.57 ± 13.62	F (0.854)	0.466
	Comfort in faith	45	48.37 ± 10.76		
	Only during important moments of daily life	22	52.77 ± 15.82		
	Irreligious	12	52.75 ± 13.40		
History of Personal Trauma	Physical assault	21	51.52 ± 15.87	F (0.639)	0.671
	Witness Patient Violence	44	53.45 ± 9.94		
	Verbal abuse	35	49.14 ± 9.27		
	Sexual Harassment	6	51.83 ± 17.05		
	Bullying	60	49.58 ± 15.10		
	Never	15	52.80 ± 16.52		
Trauma Case Load	Less than 50	113	49.44 ± 14.30	F (7.008)	0.001
	50–100	59	51.72 ± 9.62		
	100–200	9	65.88 ± 10.78		
Organisational Support	Excellent	45	45.75 ± 13.20	F (2.807)	0.027
	Very Good	24	54.00 ± 14.79		
	Good	54	51.24 ± 12.86		
	Fair	16	54.87 ± 15.32		
	Poor	42	53.14 ± 10.62		
Relationship with Colleagues	Excellent	47	45.85 ± 12.46	F (2.846)	0.026
	Very Good	44	54.34 ± 14.75		
	Good	75	51.66 ± 12.62		
	Fair	3	55.00 ± 8.18		
	Poor	12	53.83 ± 10.25		

t: Independent t test; F: ANOVA test

#### Independent factors of secondary traumatic stress among ED nurses

Multiple linear regression shows that gender (*p* = 0.001), years of experience (*p* = 0.005), marital status (*p* = 0.013), and trauma case load (*p* = 0.007) were the independent factors of the STSS among ED nurses, see Table 6.

**Table 6:** Multiple linear regression of independent factors of Secondary Traumatic Stress among ED nurses

Factor	Variable	B	Exp(B)	Sig.	95% C.I. for EXP(B)
Age		0.101	0.043	0.693	-0.404-0.606
Gender	Male	Reference			
	Female	6.698	0.254	0.001	2.719–10.677
Years of experience		-0.610	-0.246	0.017	-1.111–0.109
Marital Status	Unmarried	Reference			
	Married	4.561	0.171	0.030	0.459–8.662
Educational level	Diploma	-0.763	-0.025	0.795	-6.569-5.042
	Bachelor	2.952	0.110	0.288	-2.517- 8.421
	Master	Reference			
Trauma Case Load	Less than 50	Reference			
	50–100	3.133	0.111	0.118	-0.806-7.072
	100–200	14.487	0.239	0.001	6.099–22.875
Organisational Support	Excellent	Reference			
	Very Good	6.733	0.173	0.032	0.603-12.863
	Good	1.595	0.055	0.605	-4.484-7.675
	Fair	4.611	0.099	0.259	-3.430-12.652
	Poor	0.494	0.016	0.891	-6.622-7.609
Relationship with Colleagues	Excellent	Reference			
	Very Good	4.379	0.142	0.138	-1.428-10.186
	Good	2.195	0.082	0.483	-3.965-8.355
	Fair	7.039	0.068	0.352	-7.854-21.932
	Poor	4.610	0.087	0.323	-4.571-13.790

R: 0.574; R^2^: 0.330; Adjusted R^2^: 0.264

### Qualitative results

#### Sociodemographic characteristics of nurses participated in qualitative phase

In a sample of 10 nurses included in the interviews, the mean age was 31.7 years, with an average professional experience of 9.5 years. The educational backgrounds among the nurses are diverse, with 6 holding Bachelor’s degrees, 3 possessing Diplomas, and 1 having a Master’s degree. The group was predominantly female, consisting of 8 females and 2 males. Regarding marital status, the distribution was mixed: 6 were married, 3 were single, and 1 was divorced. Among participants, five reported experiencing high levels of STS, while the other five reported low levels of stress. This diversity provided a more comprehensive understanding of the STS experience and the various factors influencing its manifestation (Table 7).

**Table 7:** Demographic information for nurse who were interviewed

Nurses number		Age	Gender	Marital status	Education	Experience	STS Scores
Nurse 1		27	Female	Married	Diploma	7 Y	High
Nurse 2		25	Female	Single	Bachelor	3 Y	High
Nurse 3		29	Female	Married	Bachelor	5 Y	High
Nurse 4		40	Male	Married	Bachelor	15 Y	High
Nurse 5		25	Female	Married	Bachelor	3 Y	High
Nurse 6		35	Female	Single	Master	11 Y	Low
Nurse 7		42	Female	Divorced	Diploma	20 Y	Low
Nurse 8		33	Female	Married	Diploma	12 Y	Low
Nurse 9		29	Male	Single	Bachelor	6 Y	Low
Nurse 10		32	Female	Married	Bachelor	10 Y	Low

#### Findings of the interviews

Five themes emerged from the qualitative interviews: ED Characteristics: Dual Impact on STS, Emotional Resonance and Vulnerability, Personal Life Stressors, The ability to cope and Social support.

### Theme 1: ED characteristics: dual impact on STS

Some nurses reported that working in the ED made them experience fewer physiological and psychological problems when providing care to patients, especially those nearing death. The nurses indicated that since their transfer to the ED, they haven’t had to establish close bonds with patients, as they care for them for a short time. In contrast, participants reported that departments like the dialysis unit, where patients need ongoing treatment over extended periods, require nurses to engage in more prolonged relationships with their patients. This dynamic presents unique emotional challenges, as observed in other specialized units like the isolation ward, highlighting the diverse impacts of different nursing environments on healthcare professionals’ well-being.

“I developed a strong connection with a patient when I was working in the ward, and I was profoundly impacted by their death. Now, in the ER, I am unable to establish relationships with patients, regardless of my desire to do so.” (Nurse 6).

“I used to work in the isolation sections and built long-lasting relationships with many patients who stayed there. I was deeply affected if something happened to them. Now I feel less attached to the patients since transferring to the ER’’ (Nurse 8).

“I know a colleague who works in the dialysis unit and cries every time a patient dies. Even though he is not typically sensitive, he finds it difficult to cope with these losses.” (Nurse 9).

On the other hand, some nurses find it challenging to detach emotionally from their work, highlighting the intricate nature of nursing care where emotional bonds are fundamental to the profession. This sentiment is encapsulated in the words of one nurse:

“I have encountered several shocking events that continue to weigh heavily on me. My colleagues advise professional detachment; however, I cannot comply because I believe that our emotions as nurses are essential to delivering true care” (Nurse 3).

“We will continue to experience stress, and it’s unlikely and challenging to completely separate our emotions from our work as nurses.” (Nurse 4).

Responses from new nurses revealed a common struggle with distressing experiences at work. One nurse shared their difficulty in staying emotionally detached, as advised by her nurse’s colleagues, because she felt that connecting emotionally is crucial for providing proper care.

Additionally, some nurses have reported being more profoundly impacted by traumatic situations due to feelings of guilt and hopelessness. These emotions stem from the perceived low quality of care they are able to provide, which is linked to the excessive burdens and demands characteristic of ED environments

“Occasionally, I feel distressed by the thought that I could have provided more care to certain patients if I had not been so overwhelmed with other responsibilities.” (Nurse 2).

Another nurse described the challenging nature of work in the ED, particularly for those handling critical and life-threatening situations. She mentioned the difficulty of dealing with high-pressure scenarios such as resuscitations and witnessing patient deaths.

“Handling cases like resuscitations and witnessing deaths in daily bases has been tough. It’s these kinds of intense, acute events that really stick in my mind” (Nurse 5).

### Theme 2: emotional resonance and vulnerability in nursing

The emotional resonance and vulnerability experienced by nurses significantly shape their professional practice and the care they provide to patients. This theme encompasses the profound impact of personal experiences, such as parenthood, and inherent personality traits, like anxiety, on nurses’ interactions with patients and their well-being. Nurses report an intensified emotional connection with patients that mirrors their own life experiences, such as the empathy felt by parent-nurses towards pediatric patients or the poignant reminder of lost loved ones when caring for elderly patients.

“In every child that comes into the ER, I see the image of my own child. Sometimes, I choose not to work with these young patients and instead ask my colleagues to take over their care.” (Nurse 1).

Another nurse also feels a strong connection to senior patients, reminiscent of her late father. She experiences a deep emotional bond with these patients says:

“Each senior man with a white beard who arrives in the ED holds a special place in my heart, reminding me of my father who has passed away—may he rest in peace. When something happens to them, it makes my heart melt with grief, and it feels as if I am experiencing the loss of my father all over again,” (Nurse 2).

Additionally, certain personality traits, such as a tendency towards anxiety, can increase vulnerability to STS. Nurses with these traits may be more prone to internalizing and reflecting on the traumatic experiences of others.

“I’ve always been a bit of a worrier. Lately, I catch myself thinking and dreaming about my patients’ struggles even after my shift is over.” (Nurse 4).

“Everything I see in the hospital reflects on me at home. When my children fall ill, I live in terror that something will happen to them like what happened to a patient I saw in the hospital. There was a child who developed a fever, then had seizures and complications that might impair them for life, even though they were a normal child before. I have become obsessed and fearful that something similar will happen to my children. My husband gets upset about my excessive concern for our children, even in minor cases.” (Nurse 3).

The stress of working in high-pressure environments like the ER, compounded by the emotional intensity of caring for pediatric patients, can lead parent-nurses to become overly vigilant or anxious about their own children’s well-being, even in minor situations. This excessive concern, a possible manifestation of STS, can strain family relationships, as illustrated by instances where a spouse, such as a husband, becomes upset over what is perceived as unnecessary worry. This quotation indicates that the stress from work can spill over into their personal life, leading to a cycle where the stress from one domain exacerbates the challenges in the other.

### Theme 3: personal life stressors

External stressors in one’s personal life, such as family issues, health problems, financial challenges, or other personal difficulties, can compound the stress experienced at work. When personal resources are already strained, the additional burden of STS can be even more impactful.

“Dealing with my own family problems and money issues at home makes the stress from my job even harder to handle.” (Nurse 4).

” I am currently facing a significant emotional exhaustion and find myself unable to manage additional stressors. Following my diagnosis with Multiple Sclerosis, I am grappling with persistent feelings of fear and uncertainty about the future on a daily basis.” (Nurse 5).

### Theme 4: the ability to cope

Some nurses effectively manage their emotions during patient care, employing strategies to maintain a professional demeanor in emotionally charged and potentially stressful situations, such as when delivering distressing news to families about the loss of a loved one, a diagnosis, or a tragic accident

“I requested the doctor to be the one to convey the difficult news to the patient’s family because I find it emotionally challenging. It was particularly distressing for me when one of my patients tragically lost both of their legs in a car accident, and I felt unable to communicate this heart-breaking situation to their family’’ (Nurse 10).

‘’Now, after all these years, I have developed a thick skin that shields me from the intrusion of sadness into my body” (Nurse 9).

The quotation underscores how certain nurses cultivate resilience over time to handle stressors, aptly described as “developing a thick skin.” This phrase metaphorically signifies the establishment of emotional boundaries or wall, enabling nurses to fulfil their responsibilities without permitting emotional distress, stemming from continuous exposure to traumatic situations, to affect them deeply.

### Theme 5: social support

Some nurses reported that they have a strong support network within the workplace, which helps nurses cope with STS.

“One of my colleagues experienced a deeply distressing event when her brother passed away in room number 3. As a result, she has developed severe symptoms of distress whenever she is required to enter that room. Since then, we have rallied together as a team to provide her with emotional support and assistance in managing her difficulty. Additionally, we have volunteered to handle her assignments if they happen to be in that room.” (Nurse 10).

The influence of social support was clearly evident, as some nurses, who preferred to avoid working with children after becoming parents themselves, received support from their colleagues by taking those assignments from them. Additionally, a nurse who had faced a traumatic incident in a particular emergency room was supported by the supervisory team, which accommodated her by scheduling shifts in a different room. Others received assistance from doctors in communicating sensitive news to patients or their families, which are measures aimed at reducing STS. These varied forms of support play a crucial role in alleviating the impact of STS among nursing staff in ED.

## Synthesis and integration

In our study’s quantitative phase, we observed significant variations in stress scores among nurses. Qualitative interviews revealed that this variation is partly due to the unique dynamics of the ED. Some nurses experienced less stress, attributing it to the brief and less emotionally involved nature of patient care in the fast-paced ED environment. In contrast, others reported higher stress levels, particularly those in critical care roles within the ED, who face high-pressure situations like resuscitations and patient deaths. These findings highlight the complexity of stress factors in emergency medical settings.

In the quantitative phase, we observed a correlation between relationships with colleagues, organizational support, and heightened levels of STS among ED nurses. The qualitative insights revealed that this relationship is multifaceted. Nurses frequently relied on their colleagues for emotional and practical support in managing the high-stress environment of the ED. This involvement included nurses sharing patient care responsibilities to alleviate individual stress burdens and actively seeking advice on strategies, like maintaining professional detachment to lessen emotional involvement with patients. Additionally, nurses often sought the assistance of doctors in communicating sensitive information or ‘breaking news’ to patients and their families, as a means to manage the emotional impact of such interactions. This collaborative approach within the healthcare team plays a crucial role in the overall management of STS in the demanding environment of the ED, highlighting the need for comprehensive support systems within healthcare settings.

Quantitatively, the higher incidence of STS among married nurses could be attributed to the additional responsibilities and pressures that often come with marital and familial commitments. This observation aligns with the qualitative accounts where nurses reported that external stressors in their personal lives, such as family issues and health problems, exacerbate the stress experienced at work. It is also reasonable to infer that many married nurses are also parents, and this role can significantly influence their emotional and psychological responses, especially in their professional interactions involving children. Parenthood inherently brings a deeper empathy and sensitivity towards children, which could intensify the emotional experiences of nurses when caring for pediatric patients or dealing with pediatric emergencies. Given the higher number of children presenting to these settings, adds an important dimension to the stress experienced by nurses, especially those who are parents. Healthcare institutions should be mindful of these dynamics and consider flexible work arrangements, comprehensive mental health support, and resources that address both work-related and personal stressors.

In the initial phase of our study, examining the relationship between the number of children and STS among nurses did not reveal a significant correlation. However, being a parent was reported to be related to higher STS. Subsequent qualitative insights indicated a notable trend: nurses who are parents, especially mothers, experienced an enhanced emotional impact when caring for pediatric patients. This underscoring the complex interplay between personal and professional roles in healthcare settings.

We noticed that the avoidance score was high when measuring STS, aligning with qualitative findings that reported the common coping strategy among nurses is the avoidance of stressors to preserve emotional stability. For instance, several nurses, particularly after becoming parents, chose to avoid working with pediatric patients. Additionally, a nurse who experienced a traumatic event in a specific area received support from the supervisory team, who responded by reassigning her to different areas. Furthermore, some nurses were assisted by doctors in delivering sensitive news to patients and their families, thus mitigating the potential trauma. This pattern of avoidance as a coping mechanism underscores the need for comprehensive strategies to address the complex emotional challenges faced by nursing staff in various healthcare settings. The increased caseload leading to heightened STS aligns with qualitative findings that reported high caseloads often result in limited time and resources for each patient. Nurses may feel that they are not providing the level of care they aspire to, which can lead to feelings of guilt and hopelessness. This emotional response is particularly pronounced in cases with poor outcomes, despite the nurse’s best efforts.

## Discussion

The integration of quantitative and qualitative findings in this study provides a multifaceted analysis of the experience of STS and how its levels are influenced by several factors. From the findings of this study, it is evident that the STSS prevalence levels among ED nurses in Saudi Arabia are high—95% of ED nurses experience STS with different severity. This is in accordance with Ratrout [11], who reported an approximately similar prevalence of STS (94%) among ED nurses. In this study, more than half of ED nurses experienced high to severe levels of STS, with the majority of them reporting at least one symptom of STS. The obtained findings are similar to those of previous studies [10, 11, 33]. A critical analysis of the existing literature indicates that there is a prevailing high exposure to and risk of STS and PTSD among ED nurses. This can be explained by the nature of the nurses’ jobs and responsibilities [34]. The ED is mandated to care for emergency situations, such as injuries caused to accident victims, unexpected death, and violence [35]. In particular, their constant interaction with new death experiences of patients in the ED with significant injuries and pain, and even the loss of patients to death under their care, is a possible trigger for developing STS [36]. This exposure necessitates the implementation of targeted support systems and resilience-building programs within healthcare settings.

Our findings indicate that some nurses in the ED experienced lower levels of STS due to a diminished attachment to patients, attributing this to the transient and less emotionally involved nature of patient care inherent in the fast-paced ED environment. This detachment is partly due to the high acuity and urgency of cases encountered in the ED, where the primary focus is on providing immediate care. Patients often do not stay in the ED for extended periods; they are either quickly transferred to other departments for further treatment or discharged. This dynamic environment, characterized by brief interactions and the rapid turnover of patients, limits nurses’ ability to establish the kind of long-term relationships that might develop in less acute settings, such as long-term care units. Conversely, our study also revealed that certain nurses, particularly those involved in critical care roles within the ED, reported experiencing higher levels of stress. This increase in stress is attributed to the high-pressure situations they frequently face, such as performing resuscitations and managing patient deaths. These findings illuminate the varied impact of the ED work environment on nurses’ experiences of stress and emotional involvement with patients. This highlights the need for tailored interventions and support strategies in the ER, acknowledging both the challenges and potential positive aspects of this unique setting. Such targeted support is essential for effectively helping nurses manage STS.

In this study, it was evident that ED nurses suffer considerably from stress avoidance, intrusion, and arousal symptoms (rated as moderate and above) when measured through the lens of STS which was constant with a study conducted in Greek and reported similar findings [33]. Among the three subscales, avoidance scored the highest. This result was clearly evident in the avoidance behaviors that nurses utilize to cope with STS, as observed in the qualitative phase of the study. This aligns with the findings of Qian [37], who reported similar observations. The findings suggest that healthcare institutions should invest in targeted training programs that focus on emotional resilience and stress management. This training could help nurses develop healthier coping mechanisms beyond avoidance.

The results showed that the most reported symptoms were psychological stress, difficulty sleeping, foreshortened future, diminished activity level, hypervigilance, and irritability, respectively. These symptoms were also reported in Ireland by Duffy et al. [38] and in USA by Dominguez-Gomez and Rutledge [39]. Nurse managers and organisations should create effective strategies to reduce and manage such symptoms and prevent their consequences.

Being female nurses was associated with increasing the levels of STS. This finding was similar to Civljak et al. [40] Ramatsipele [41] and Dominguez-Gomez and Rutledge’s [39] and contrasted with those of Mary Pappiya [42]. Although these studies were conducted in USA, the variation between them might be related to the variation in the criteria used to measure STS [11]. The existing literature reported that female nurses are more prone to stress because of the multi-role and responsibilities associated with being a wife or mother [43]. In addition, female nurses in Saudi Arabia expose to night working shift that consider difficult and, culturally unacceptable and provide more stressful situation for them [44, 45]. Given that the nursing workforce comprises mostly female, gender-specific interventions to reduce STS is required. Therefore, our findings suggested that married nurses may be more likely to demonstrate higher levels of STS, which was consistent with the results of Lee et al. [46] and contrasted with those of Ramatsipele [41]. A popular explanation is that the higher stress can be a consequence of the role of married nurses, which involves complex and multiple responsibilities to fulfil, such as being a parents, husband/wife, housekeeper, and employee, which might increase the level of perceived stress among them [47, 48]. Contrary, it has been reported by Jiang et al., that being married and having a stable partner could be a source of support to reduce stress [49]. However, Robles stated that being married is not an advantage if the quality of marriage is low [50].

Further, this study revealed that the levels of STS are lower in cases where nurses have a higher number of years of experience. According to Labrague [51], nurses with lower number of years of experience had significantly higher stress due to the fear of medical errors, lack of assessment skills, and fear of occupational injuries [51]. Experienced nurses deliver higher-quality care and possess the ability to adapt to uncertain, everyday situations in dynamic environments like the ED and its various challenges. These seasoned nurses can cope effectively with stress and offer social support to both their vulnerable colleagues and new nurses who are still learning to confront STS. Further research on experienced nurses’ strategies underscores the importance of structured mentorship programs to facilitate knowledge transfer and stress management, enhancing workplace support and efficiency.

The current study revealed that an increased trauma caseload significantly increases STS. Several studies have found a significant positive association between STS and the number of trauma cases admitted to the ED [52, 53]. According to McCann and Pearlman, hearing or learning about a traumatic event can induce STS [54]. In addition, reinforcement of nurses with coping strategies should be planned to help them to improve mental wellbeing, decreases stress and improve their resilience [55]. So that, psychological support and assistance from the healthcare providers should be provided for nurses to improve their working conditions [56]. Administrators and policymakers should encourage reasonable client caseloads, which is important to reduce STSS among ED nurses [57].

This study also found that the experience of STS among nurses of different races and ethnicities differs significantly, although it was not significant after we performed the regression analysis. Cultural differences, traditions, beliefs, expectations, and behaviors can influence the level of reported stress among nurses. According to Aldwin, the cultural context shapes the types of stressors that an individual is likely to experience and the manner in which these stressors are perceived, understood, and dealt with [58].

Although extant literature has reported that work-related factors—such as weekly working hours, career rank, salary, shift work, and organisational support—played a significant role in the prevention or occurrence of STS among professionals [38], our findings show no such influences. To conclude, STSS may have a few limitations. One limitation of the study was the inability to contact certain eligible participants for the interview phase, as they did not provide their contact details in the online survey during the initial quantitative phase. We interviewed a purposeful sample of ED nurses with varying STSS scores. If a nurse was not interested in participating in the subsequent phase or had not provided contact information, we recruited those with the next highest or lowest scores, which might not have been the ideal choice for the study’s purpose. Another limitation is that during the interviews, it was noted that some nurses held misconceptions about STS, frequently focusing on the general stress and challenges of working in the ED instead. In these instances, the researcher provided clarification on the concept of STS and guided the participants back to the central topic of the interview. The researcher clarified the concept of STS and steered the participants back to the intended focus of the interview. An additional limitation of our study is the subjective nature of certain data points, such as trauma case load, organizational support, and history of trauma. These variables depend on participants’ experiences and may introduce bias into the study results. However, we mitigated this by quantifying these data using standard scales, which enhanced the reliability, comparability, and objectivity of our data analysis.

## Conclusion

In summary, the study has demonstrated an insight into the nature of STS and its impact on nursing professionals. These messages underscore the complex dynamics of STS in healthcare settings and offer guidance for addressing this pervasive issue. Firstly, the study reveals the high prevalence of STS among ED nurses, with a significant portion experiencing severe levels of stress. This underscores the emotionally taxing environment of emergency care and the urgent need for targeted interventions to support the mental health and well-being of these essential healthcare workers. Secondly, the study identifies key demographic and occupational factors associated with higher levels of STS, including gender, marital status, years of experience, and trauma caseload. These insights can inform targeted interventions, such as providing additional support for female nurses, those with greater familial responsibilities, or staff handling a high volume of trauma cases. Thirdly, the research highlights the dual impact of the ED environment on STS, showing how the fast-paced, high-pressure setting can both mitigate and exacerbate stress levels. Nurses in the ED may experience reduced emotional attachment due to brief patient interactions, potentially lowering STS. Conversely, the critical nature of care in the ED, involving life-threatening situations and patient deaths, significantly heightens the risk of STS. This dichotomy emphasizes the need for nuanced support strategies that address the unique challenges of the ED setting. Moreover, the study points to the profound influence of personal factors, such as family-linked empathy and personal vulnerabilities, on nurses’ experiences of STS. Nurses who are parents or have strong personal connections to their patients may find these emotional bonds intensifying their stress. This finding suggests the importance of considering individual nurse’s backgrounds and personal lives when developing support and intervention programs. Additionally, the investigation into coping mechanisms and social support systems within the workplace reveals their critical role in mitigating STS. Strategies that promote professional detachment while fostering a supportive team environment can help nurses manage the emotional demands of their work more effectively.

In conclusion, the study offers vital perspectives on the challenges ED nurses face regarding STS. Healthcare institutions should implement regular training on stress recognition and coping strategies, establish peer support programs, and provide accessible professional mental health support. Policies on workload management are essential to prevent nurse overload and ensure periodic rotations to less intense environments. Enhancing the work environment with quiet spaces for breaks and ergonomic improvements can also reduce stress. Additionally, leadership training should focus on supportive practices that foster a positive work culture, complemented by systems for regular mental health assessments and resilience-building programs to equip nurses with tools to manage and mitigate the impacts of STS effectively.

### Electronic supplementary material

Below is the link to the electronic supplementary material.

Supplementary Material 1

## Data Availability

The datasets used and/or during the current study are available from the corresponding author on reasonable request.

## Abbreviations

ED: Emergency department
STS: Secondary traumatic stress
STSS: Secondary traumatic stress scale
DSM-5: The Diagnostic and Statistical Manual of Mental Disorders 5th Edition
PTSD: Post-traumatic stress disorder
IRB: Institutional Review Board
GRAMMS: The Good Reporting of a Mixed Methods Study
