# Supplementary material for: Exposure to secondary traumatic stress and its related factors among emergency nurses in Saudi Arabia: a mixed method study
Source: BMC Nurs. 2024 May 18;23:337. doi: 10.1186/s12912-024-02018-4 (PMC11102619; doi:10.1186/s12912-024-02018-4)
Supplement: Supplementary file 1 — Supplementary Material 1 [file 12912_2024_2018_MOESM1_ESM.docx]

**The interview guide (Supplementary 1)**

1. **Describing Stressful Incidents:**

Can you describe a specific incident in your nursing career that was particularly stressful or emotionally challenging?

**Probes**: What were the key factors that made this incident stressful? How did you feel during and after the situation?

1. **Factors Influencing STS:**

What factors in your work have exacerbated or alleviated the traumatic stress you experience?

Are there any specific factors or aspects of your work environment that you believe contribute to higher or lower levels of STS (explain)?

**Probes**: Can you give examples of situations or conditions that intensified your stress? What actions or changes have helped reduce your stress?

1. **Work Environment and STS:**

In your opinion, are there aspects of your work environment that contribute to higher or lower levels of STS?

**Probes**: How does the work culture or management style impact your stress levels? Are there physical or organizational aspects of your workplace that affect STS?

1. **Improving Well-Being and Mental Health:**

What measures do you think could be implemented to improve the well-being and mental health of nurses facing frequent STS?

**Probes**: Can you suggest any specific policies or support systems? How can these measures be effectively integrated into the nursing environment?

1. **Personal Experience with Overwhelming STS:**

Can you recall a time when you felt particularly overwhelmed by STS? How did you handle this, and what kind of support did you seek or receive?

**Probes**: What coping strategies did you find most effective? How responsive and helpful was the support you received?

1. **Managing STS Professionally:**

How do you manage or deal with STS in your professional capacity?

**Probes**: Are there any routine practices or habits you follow to mitigate stress? How do you balance your professional responsibilities with self-care?

1. **Reflecting on Personal Growth:**

Looking back at your experiences with STS, how do you think they have influenced your personal and professional growth?

**Probes**: What lessons have you learned from dealing with stressful situations? How have these experiences shaped your approach to nursing?

1. **Support Systems and Resources:**

What type of support systems or resources do you believe are essential for nurses dealing with STS?

**Probes**: Are there specific types of training or educational resources that you find valuable? How can healthcare institutions better support nurses in this regard?

1. **Balancing Work and Personal Life:**

How do you balance the demands of your nursing career with your personal life, especially when dealing with STS?

**Probes**: What strategies do you use to maintain this balance? How do you prioritize your mental and emotional health?
